# Increasing retractions of meta-analyses publications for methodological flaw

**DOI:** 10.1186/s13643-021-01822-2

**Published:** 2021-10-08

**Authors:** Chia-Yun Chen, Yi-No Kang, Ken N. Kuo, Paul Glasziou, Kee-Hsin Chen

**Affiliations:** 1 Medical School, College of Medicine, Taipei Medical University, Taipei, Taiwan; 2 Cochrane Taiwan, Taipei Medical University, Taipei, Taiwan; 3 Evidence-Based Medicine Center, Wan Fang Hospital, Taipei Medical University, Taipei, Taiwan; 4 Research Center of Big Data and Meta-analysis, Wan Fang Hospital, Taipei Medical University, Taipei, Taiwan; 5 Institute of Health Policy and Management, College of Public Health, National Taiwan University, Taipei, Taiwan; 6 Department of Orthopaedic Surgery, National Taiwan University Hospital, Taipei, Taiwan; 7 Institute for Evidence-Based Healthcare, Bond University, Level 4, Building 5, Faculty of Health Sciences and Medicine, 14 University Drive, Robina, QLD, 4226 Gold Coast, Australia; 8 Post-Baccalaureate Program in Nursing, College of Nursing, Taipei Medical University, No. 250, Wu-Xing Street, 110 Taipei, Taiwan; 9 Center for Nursing and Healthcare Research in Clinical Practice Application, Wan Fang Hospital, Taipei Medical University, Taipei, Taiwan; 10 Evidence-based Knowledge Translation Center, Wan Fang Hospital, Taipei Medical University, Taipei, Taiwan

## Abstract

Purpose of this letter was to explore the trends regarding methodological flaws of systematic review and meta-analyses (SRMAs) based on retraction notes in the past decades, and the categories of reasons for the retractions. Content analysis with descriptive statistics, Cochran *Q* test, and multinomial logistic regression were used. Based on 187 records of retracted SRMAs, retraction announcements can be categorized into academic ethical violation, methodological flaw, and writing or reporting problem. The numbers of academic ethical violation were significantly higher than those with methodological flaw (*z* = 3.51; *p* < 0.01) or writing problem (z = 8.58; *p* < 0.001). The numbers of methodological flaw were also higher than that with writing problem (*z* = 6.47; *p* < 0.001). Moreover, an increased proportion of methodological flaw was observed since 2006, and the retraction year was significantly associated with increased proportion of methodological flaw when academic ethical violation as the reference group.

**Supplementary Information:**

The online version contains supplementary material available at 10.1186/s13643-021-01822-2.

## Background

There were significant increases in the publications of systematic reviews and meta-analyses (SRMAs) during past decade, but some meta-analyses seemed to be flawed and non-informative synthesizing [1, 2]. Mass productions of SRMAs raise concerns [1–3], and flaws of SRMAs have received scholarly attention [1]. Inappropriate data syntheses may lead to research waste [4], and result in retraction due to unreliable findings and conclusions. It is the purpose of this letter to review the methodological flaws of SRMAs retracted and the retraction notes for the understanding of methodological flaws in syntheses.

## Methods

The present letter searched references from three databases using relevant keywords before June 2021 (Additional file 1). Two authors independently double-checked the eligibility of references, information extraction, and classifications of reasons for retractions. They extracted authors’ name, retraction year, journal, and the reasons for retractions. There was no predefined frame for the classification of the retraction reasons, and the categories were constructed through content analysis. Since some retraction notes concurrently covered two or more categories, the classifications were recorded as multiple paired binary variables. R version 4.0.3 was used for analysis. Cochran *Q* test with adjusted *P* value was done for comparing counts among categories of the reasons of retractions using command “pairwiseMcNemar” in “RVAideMemoire” package. To test whether retraction year was accounted for the difference in reasons for retractions, multinomial logistic regression was further performed using the command “multinom” in “nnet” package.

## Results

**Fig. 1:**
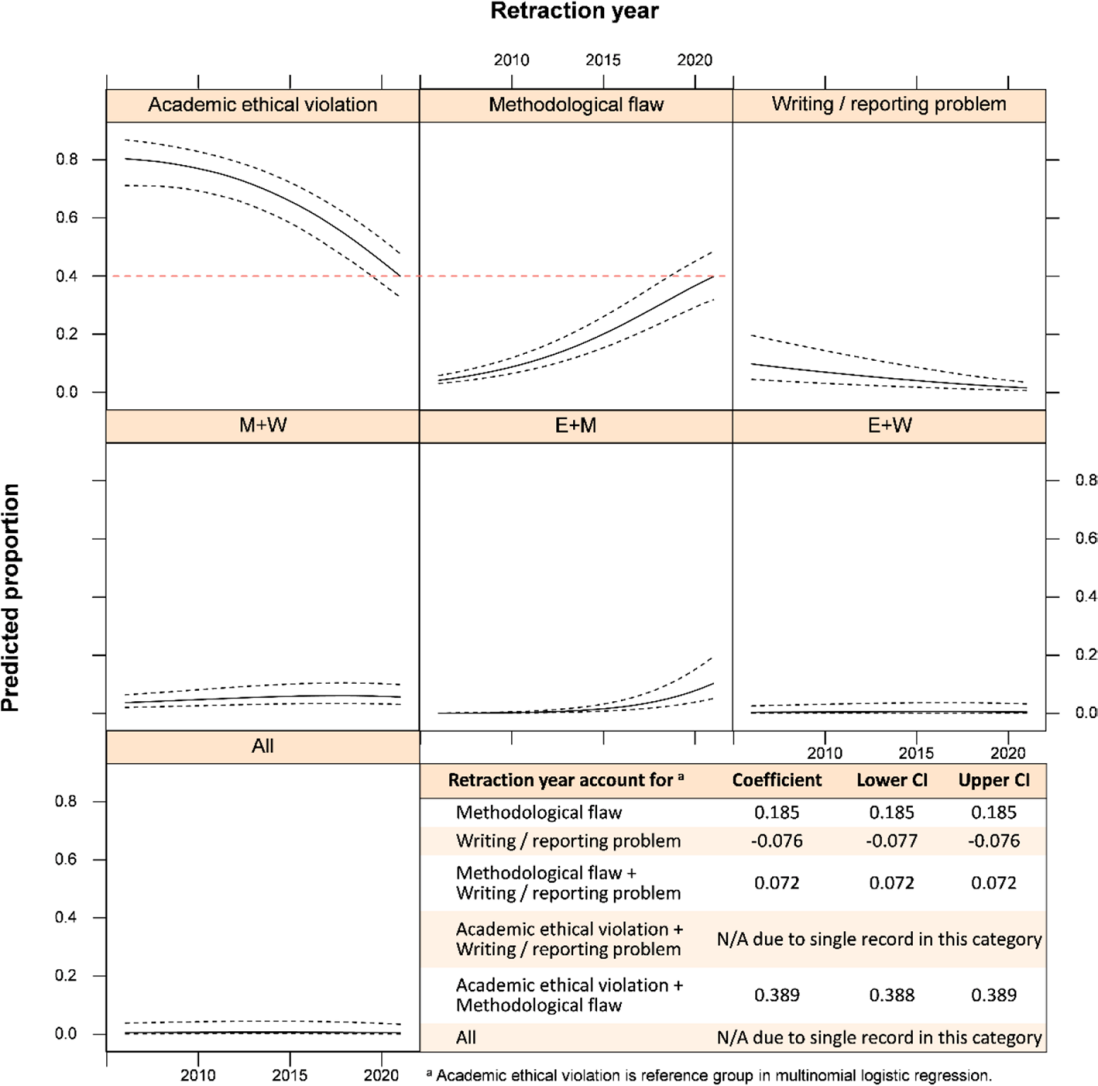
Multinomial logistic regression of retraction year on reasons of retraction. CI, 95% confidence interval; E, academic ethical violation; M, methodological flaw; W, writing or reporting problem

A total of 198 retracted meta-analyses were identified (Additional file 2), but 11 retracted records were not included in the present analysis because there was no retraction reason (*i* = 6) or special cases (*i* = 5). In those five special cases, the SRMAs were not problematic because one or some of the original researches in those syntheses were retracted after the SRMA were published. Based on the remaining 187 records, retraction announcements can be categorized into academic ethical violation, methodological flaw, and writing or reporting problem (Additional files 3 and 4). The most common reason for retractions was academic ethical violation (*i* = 118, 69.82%) followed by methodological flaw (*i* = 72, 42.60%), and writing or reporting problem (*i* = 19, 11.24%; Additional file 5). The numbers of academic ethical violation were significantly higher than those with methodological flaw (*z* = 3.51; *p* < 0.01) or writing problem (*z* = 8.58; *p* < 0.001). The numbers of methodological flaw were also higher than that with writing problem (*z* = 6.47; *p* < 0.001; Additional file 6). Moreover, it is observed that there was an increased proportion of methodological flaw since 2006. In the other way, increased proportion of methodological flaw was significantly associated with the retraction year when academic ethical violation was used as the reference group (Fig. 1).

## Discussion

The most common reasons for retractions of meta-analyses were academic ethical violation and methodological flaw. Academic ethical violation for retraction of SRMAs publications is gradually decreasing, while methodological flaw for retractions is increasing in the recent years, particularly after 2015. These trends may be due to rapid development and maturing of methodology in evidence synthesis. For instance, methodology of SRMA becomes more familiar after 2009 due to newer version of the Cochrane Handbook and the PRISMA guideline [5, 6]. With the increased understanding of methodology of SRMA in academic society, scholars in recent years may identify methodological flaws in SRMAs more than before. Involvement of nonconflicting meta-analysis experts in research team or inviting nonconflicting meta-analysis experts to take part in the peer review are still important because experts in SRMA may improve the quality control of meta-analysis publications [3]. Though the official announcement of retraction is objective, however, it is difficult to understand the complexity of the context behind the words. Therefore, the real reason behind the official statement may be limited in this study. Further qualitative or mixed method studies are foreseeably valuable in the future.

## Supplementary Information

**Additional file 1.** Database and search strategy.

**Additional file 2.** Flow diagram of study selection.

**Additional file 3.** Examples of category of problems.

**Additional file 4.** Reference characteristics of retracted meta-analyses before June 2021 (i=198).

**Additional file 5.** Doughnut plot of clarity of reasons for retractions of meta-analyses.

**Additional file 6.** Plot of Cochran Q test for three categories of retraction reason.

## Data Availability

All data generated or analyzed during this study are included in this published article.

## Abbreviation

SRMA: Systematic review and meta-analysis
