# Supplementary material for: Increasing retractions of meta-analyses publications for methodological flaw
Source: Syst Rev. 2021 Oct 8;10:267. doi: 10.1186/s13643-021-01822-2 (PMC8499503; doi:10.1186/s13643-021-01822-2)
Supplement: Supplementary file 1 — Additional file 1. Database and search strategy. [file 13643_2021_1822_MOESM1_ESM.pdf]

Additional file 1 | Database and search strategy

Primary search strategy

#1. retract*

#2. meta-analysis

#3. meta-regression

#4. metaanalysis

#5. metaregression

#6. network meta-analysis

#7. dose-response meta-analysis

#8. #2 OR #3 OR #4 OR #5 OR #6 OR #7

#9. #1 AND #8

Embase (i = 210)

retract*:ti AND ('meta analysis'/exp OR 'analysis, meta' OR 'meta analysis' OR 'meta-analysis' OR 'metaanalysis' OR 'meta regression'/exp OR metaregression OR 'network meta-analysis'/exp OR 'network meta-analyses' OR 'network meta-analysis' OR 'network metaanalyses' OR 'network metaanalysis' OR 'dose-response meta-analysis')

PubMed (i = 205)

retract*[ti] AND (meta-analysis OR meta-regression OR metaanalysis OR metaregression OR network meta-analysis OR dose-response meta-analysis)

Search details in PubMed:

"retract*"[Title] AND ("meta analysis"[Publication Type] OR "meta analysis as topic"[MeSH Terms] OR "meta analysis"[All Fields] OR "meta-regression"[All Fields] OR ("meta analysis as topic"[MeSH Terms] OR ("meta analysis"[All Fields] AND "topic"[All Fields]) OR "meta analysis as topic"[All Fields] OR "metaanalysis"[All Fields]) OR ("metaregression"[All Fields] OR "metaregressions"[All Fields]) OR ("network meta analysis"[MeSH Terms] OR ("network"[All Fields] AND "meta analysis"[All Fields]) OR "network meta analysis"[All Fields] OR ("network"[All Fields] AND "meta"[All Fields] AND "analysis"[All Fields]) OR "network meta analysis"[All Fields]) OR (("dose response"[Journal] OR ("dose"[All Fields] AND "response"[All Fields]) OR "dose response"[All Fields]) AND ("meta analysis"[Publication Type] OR "meta analysis as topic"[MeSH Terms] OR "meta analysis"[All Fields])))

Web of Science (i = 356)

TITLE: (retract*) AND TOPIC: (meta-analysis OR meta-regression OR metaanalysis OR metaregression OR network meta-analysis OR dose-response meta-analysis)

Timespan: All years. Indexes: SCI-EXPANDED, SSCI, A&HCI, ESCI.
