# Supplementary figures and images for: Increasing retractions of meta-analyses publications for methodological flaw
Source: Syst Rev. 2021 Oct 8;10:267. doi: 10.1186/s13643-021-01822-2 (PMC8499503; doi:10.1186/s13643-021-01822-2)

**Additional file 2 | Flow diagram of study selection**

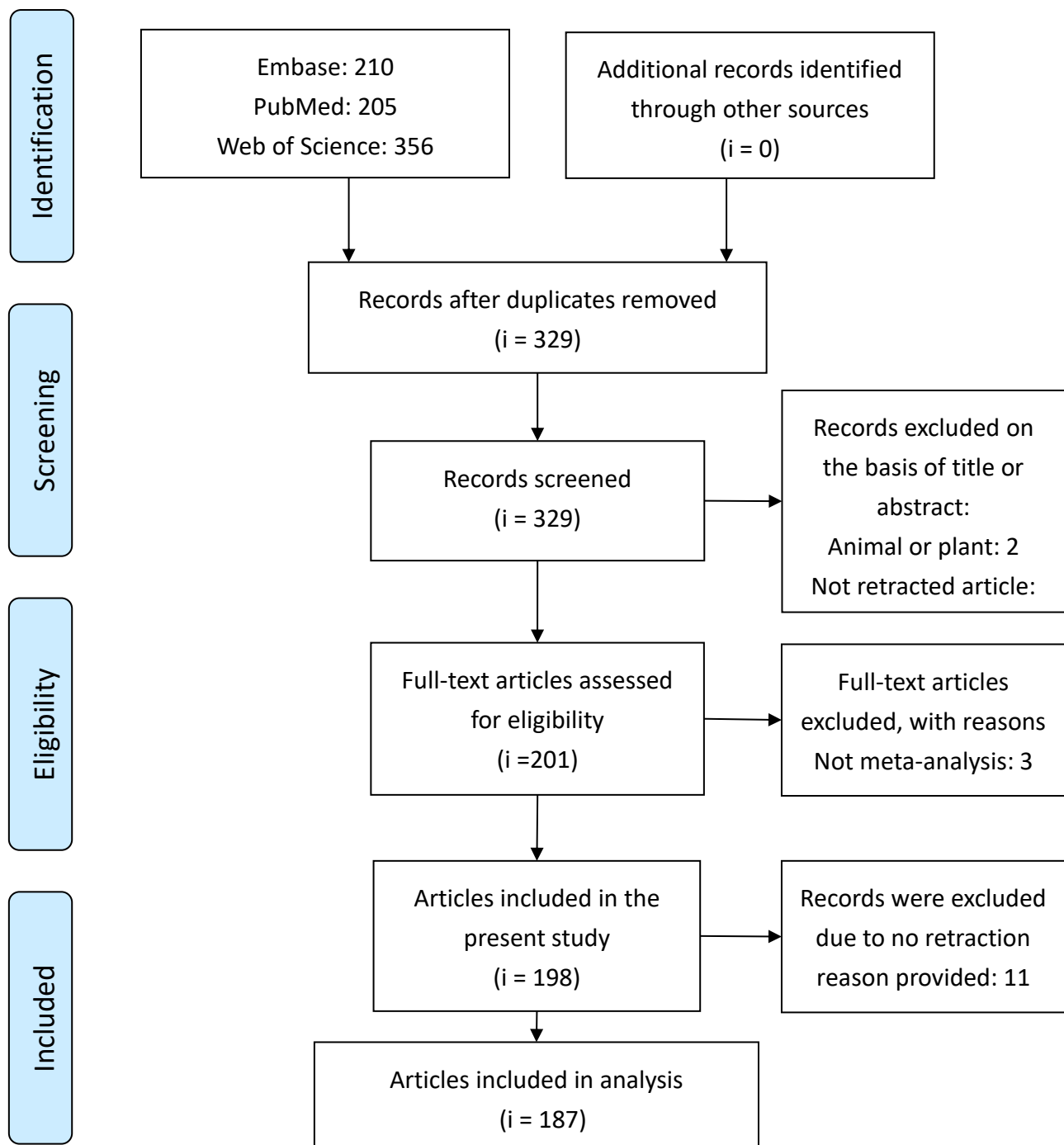

Supplement: Supplementary file 2 — Additional file 2. Flow diagram of study selection. [file 13643_2021_1822_MOESM2_ESM.pdf]
