# Supplementary material for: Increasing retractions of meta-analyses publications for methodological flaw
Source: Syst Rev. 2021 Oct 8;10:267. doi: 10.1186/s13643-021-01822-2 (PMC8499503; doi:10.1186/s13643-021-01822-2)
Supplement: Supplementary file 3 — Additional file 3. Examples of category of problems. [file 13643_2021_1822_MOESM3_ESM.pdf]

Additional file 3 | Examples of category of problems

Academic or ethical violation (69.82%)

Example 3.1: "*After a thorough investigation we have strong reason to believe that the peer review process was compromised."* – Likely fraudulent peer review

Example 3.2: "*This article is one of a series of very similar meta-analyses written by different authors that were published in 2014 and 2015, ... The articles have the same structure, with the figures in the same order. The appearance of the figures and parts of the text are also similar.*" – Likely plagiarism

Example 3.3: "*In addition, we checked the original files submitted by the authors and found that the files were edited by MedChina, a company previously alleged to be involved in the sale of articles...*" – Likely ghost authorship

Methodological flaw (42.60%)

Example 1.1: "*...parameters in the meta-analysis software were set incorrectly, leading to misleading data plots and erroneous conclusions regarding group differences.*" – Faulty analysis

Example 1.2: "*The article states in the Methods section that the analysis was done on adult in-hospital patients. However, the studies analyzed used outpatient cohorts.*" – Inappropriate pooling (involve both in-hospital patients and outpatients)

Example 1.3: "*After publication of this article…, concerns were raised about aspects of the methods applied and whether the included studies adhered to the reported inclusion and exclusion criteria...*" – Methodological flaw and problems in evidence selection

Writing or reporting problems (11.24%)

Example 2.1: "*The retraction has been agreed because the article is not yet ready for publication and an early version without revisions was published in error*." – Premature version

Example 2.2: "*The article inappropriately mentions other cancers and genes, including gastric cancer in the abstract and "breast cancer" and "IFN-γ gene" in Figure 1 in the eligibility exclusion box, whereas the article is about diabetic nephropathy (DN) and VEGF.*" – Errors in reporting

Example 2.3: "*We also found that the authors note there is publication bias, but they do not account for this in their analysis or discussion.*" – Missing in reporting
