# Supplementary material for: Increasing retractions of meta-analyses publications for methodological flaw
Source: Syst Rev. 2021 Oct 8;10:267. doi: 10.1186/s13643-021-01822-2 (PMC8499503; doi:10.1186/s13643-021-01822-2)
Supplement: Supplementary file 4 — Additional file 4. Reference characteristics of retracted meta-analyses before June 2021 (i=198). [file 13643_2021_1822_MOESM4_ESM.pdf]

Additional file 4 | Reference characteristics of retracted meta-analyses before June 2021 (i=198)

No	*First Author*	*Journal*	*Retraction year*	*Method*	*Writing*	*Ethic*
S1	Armstrong	*Gerontologist*	2020	✓		
S2	Asrani	*Medicine*	2017	✓	✓	
S3	Boccardi	*J Hum Hypertens*	2019	✓		
S4	Cao	*Molecular Neurobiology*	2015			✓
S5	Chan	*Journal of Affective Disorders*	2018	✓		
S6	Chang	*Tumor Biology*	2015			✓
S7	Chen	*DNA and Cell Biology*	2015			✓
S8	Chen	*Molecular Biology Reports*	2015			✓
S9	Chen	*Molecular Neurobiology*	2015			✓
S10	Chen	*Skeletal Radiology*	2015			✓
S11	Chen	*DNA and Cell Biology*	2015			✓
S12	Cheng	*Medicine*	2018			✓
S13	Cremonini ¥	*Aliment Pharmacol Ther*	2003	N/A	N/A	N/A
S14	da Costa	*Lancet*	2016			✓
S15	De Vecchis	*High Blood Press Cardiovasc Prev*	2020			✓
S16	Deng	*Pediatric Surgery International*	2019			✓
S17	Di Lorenzo	*Circulation*	2010			✓
S18	DiNicolantonio	*Heart*	2013	✓		
S19	Dong	*Molecular Biology Reports*	2015			✓
S20	Dong	*Molecular Neurobiology*	2015			✓
S21	Ezenwa	*Journal of Human Lactation*	2020	✓		
S22	Fialho	*Aids Care-Psychological and Socio-Medical Aspects of Aids/Hiv*	2016		✓	
S23	Fischer	*Journal of Cross-Cultural Psychology*	2019	✓		
S24	Gao	*International Journal of Geriatric Psychiatry*	2015	✓	✓	
S25	Gao	*Molecular Biology Reports*	2015			✓
S26	Gao	*Tumor Biology*	2015			✓
S27	Ghaderi	*European Journal of Clinical Pharmacology*	2019		✓	
S28	Gotink	*PLoS ONE*	2019	✓		✓
S29	Grilli	*Current Opinion in Microbiology*	2017		✓	
S30	Gu	*Archives of Physical Medicine and Rehabilitation*	2017			✓
S31	Gu	*British Journal of Psychiatry*	2020	✓		

No	First Author	Journal	Retraction year	Method	Writing	Ethic
S32	Gu	*Tumor Biology*	2015			✓
S33	Gumley	*British Journal of Clinical Psychology*	2017	✓		
S34	Guo	*Crop Science*	2006	✓	✓	
S35	Guo ¥	*Eur J Prev Cardiol*	2020	N/A	N/A	N/A
S36	Han	*Tumor Biology*	2017			✓
S37	Hao	*Molecular Neurobiology*	2015			✓
S38	He	*Tumor Biology*	2017			✓
S39	Henson	*Journal of Clinical Oncology*	2016	✓		
S40	Hoang	*Cancer Research and Treatment*	2020			✓
S41	Hofmann	*Psychiatry Res*	2018	✓		
S42	Hu	*Educational Psychology Review*	2020	✓	✓	
S43	Huang	*European Journal of Medical Research*	2015			✓
S44	Huang	*PLoS One*	2014	✓	✓	
S45	Iwamoto ‡	*Clinical Drug Investigation*	2018	N/A	N/A	N/A
S46	Iwamoto ‡	*Current Medical Research and Opinion*	2017	N/A	N/A	N/A
S47	Iwamoto ‡	*Current Medical Research and Opinion*	2017	N/A	N/A	N/A
S48	Ji	*International Journal of Ophthalmology*	2017			✓
S49	Ji	*Spinal Cord*	2015			✓
S50	Jian	*Medicine*	2019	✓		
S51	Jiang	*European Journal of Medical Research*	2015			✓
S52	Jin	*Tumor Biology*	2017			✓
S53	Kamp	*Journal of Affective Disorders*	2019			✓
S54	Li	*J Cardiothorac Surg*	2015			✓
S55	Li ¥	*J Integr Med*	2013			
S56	Li	*Journal of orthopaedic surgery and research*	2018			✓
S57	Li	*Molecular Biology Reports*	2015			✓
S58	Li	*Molecular Biology Reports*	2015			✓
S59	Li	*Molecular Neurobiology*	2017			✓
S60	Li	*PLoS One*	2018	✓	✓	
S61	Li	*Tumor Biology*	2017			✓
S62	Li	*Tumor Biology*	2017			✓
S63	Li	*Tumor Biology*	2015			✓
S64	Li	*Tumor Biology*	2015			✓
S65	Liang	*International Journal of Clinical and Experimental Medicine*	2016			✓

No	First Author	Journal	Retraction year	Method	Writing	Ethic
S66	Lin	*BioMed Research International*	2019	✓	✓	✓
S67	Lin	*Journal of Cancer Research and Therapeutics*	2017			✓
S68	Liu	*Acta Radiologica*	2019			✓
S69	Liu	*BioMed Research International*	2019			✓
S70	Liu	*International Urology and Nephrology*	2016			✓
S71	Liu	*Rheumatology*	2011	✓		
S72	Liu	*Targeted Oncology*	2016			✓
S73	López-Valverde	*Journal of Clinical Medicine*	2020	✓	✓	
S74	Luo	*Tumor Biology*	2017			✓
S75	Lv	*British Journal of Clinical Pharmacology*	2016			✓
S76	Lv	*Molecular Neurobiology*	2017			✓
S77	Lv	*Tumor Biology*	2017			✓
S78	Ma	*Tumor Biology*	2017			✓
S79	Mahboobi	*Journal of Human Hypertension*	2019	✓		
S80	Mahmoudi	*Human Vaccines and Immunotherapeutics*	2017	✓		
S81	Maria	*Resuscitation*	2019		✓	
S82	Maslej	*JAMA Psychiatry*	2020	✓		
S83	May	*International Journal of Drug Policy*	2018	✓		
S84	Mohsenzadeh	*uropean Journal of Obstetrics & Gynecology and Reproductive Biology*	2019			✓
S85	Panagioti	*JAMA Intern Med*	2020	✓		
S86	Parsaik	*Journal of Psychiatric Practice*	2018	✓	✓	
S87	Parvez ¥	*Journal of the College of Physicians and Surgeons Pakistan*	2009	N/A	N/A	N/A
S88	Poolsup	*PLoS One*	2020	✓	✓	
S89	Qian	*European Journal of Medical Research*	2015			✓
S90	Ramsay	*BMC Medicine*	2018	✓		
S91	Raslau	*Aerospace Medicine and Human Performance*	2015	✓		
S92	Ren	*Medicine*	2017	✓		
S93	Roba	*Maternal Health, Neonatology and Perinatology*	2020	✓		
S94	Shao	*Annals of Translational Medicine*	2016			✓

No	*First Author*	*Journal*	*Retraction year*	*Method*	*Writing*	*Ethic*
S95	Shen	*Journal of Cellular and Molecular Medicine*	2018			✓
S96	Siddique	*Sports Medicine*	2019	✓		
S97	Siempos	*The Lancet Respiratory medicine*	2015	✓		
S98	Soria-Gila	*Journal of Strength and Conditioning Research*	2018		✓	
S99	Stevens	*Pharm Stat*	2007		✓	
S100	Sun	*Bioscience Trends*	2016	✓		
S101	Sun	*Molecular Neurobiology*	2015			✓
S102	Sun	*The Scientific World Journal*	2020		✓	✓
S103	Sun	*Tumor Biology*	2017			✓
S104	Tan	*Alzheimers Research & Therapy*	2016	✓		
S105	Thatcher	*J Appl Psychol*	2016	✓		
S106	Tian	*Medicine*	2019	✓		
S107	Vecchis	*Interv Med Appl Sci*	2017			✓
S108	Viani	*International Journal of Radiation Oncology Biology Physics*	2013	✓		
S109	Vlok	*Am J Emerg Med*	2020			✓
S110	Wang	*Annals of Human Genetics*	2016			✓
S111	Wang	*Bmc Musculoskeletal Disorders*	2015			✓
S112	Wang	*Future Generation Computer Systems-the International Journal of Escience*	2020			✓
S113	Wang	*Gen Psychiatr*	2019	✓		
S114	Wang	*International Journal of Clinical and Experimental Medicine*	2016			✓
S115	Wang	*International Journal of Ophthalmology*	2019	✓		
S116	Wang	*Medicine*	2018	✓		
S117	Wang	*Molecular Biology Reports*	2015			✓
S118	Wang	*Molecular Biology Reports*	2015			✓
S119	Wang	*Nutrition Journal*	2018	✓		✓
S120	Wang	*PLoS One*	2016			✓
S121	Wang	*PLoS One*	2016			✓
S122	Wang	*PLoS One*	2016			✓
S123	Wang	*Tumor Biology*	2017			✓
S124	Wen ¥	*Tumor Biology*	N/A	N/A	N/A	N/A
S125	Wu	*Journal of Diabetes*	2016			✓
S126	Wu	*Molecular Neurobiology*	2015			✓

No	First Author	Journal	Retraction year	Method	Writing	Ethic
S127	Xiao	*Tumor Biology*	2015			✓
S128	Xing	*Molecular Biology Reports*	2015			✓
S129	Xing	*Tumor Biology*	2015			✓
S130	Xiong	*Ejso*	2015			✓
S131	Xiong	*Medicine*	2014			✓
S132	Xu	*European Journal of Medical Research*	2015			✓
S133	Xu	*Journal of orthopaedic surgery and research*	2015			✓
S134	Xu	*Journal of orthopaedic surgery and research*	2015			✓
S135	Xu	*Journal of Surgical Oncology*	2015			✓
S136	Xue ¥	*Tumor Biology*		N/A	N/A	N/A
S137	Yan	*Molecular Biology Reports*	2015			✓
S138	Yang	*Journal of Bone and Mineral Metabolism*	2019			✓
S139	Yang	*Journal of orthopaedic surgery and research*	2015			✓
S140	Yang	*Medical Science Monitor*	2016			✓
S141	Yang	*Molecular Neurobiology*	2017			✓
S142	Yang	*Molecular Neurobiology*	2017			✓
S143	Ye	*Eur Heart J*	2019	✓		
S144	Yin	*Journal of Dermatology*	2013			✓
S145	Yin	*Tumor Biology*	2017			✓
S146	Yu	*Molecular Biology Reports*	2015			✓
S147	Yu	*Molecular Biology Reports*	2015			✓
S148	Yuan	*Cancer Epidemiology Biomarkers & Prevention*	2019	✓		
S149	Zarychanski	*Open Med*	2010	✓		
S150	Zhai	*Tumor Biology*	2015			✓
S151	Zhang	*Int J Surg*	2017			✓
S152	Zhang	*International Journal of Clinical and Experimental Medicine*	2016			✓
S153	Zhang	*Tumor Biology*	2017			✓
S154	Zhao	*Journal of Orthopaedic Surgery and Research*	2018	✓		
S155	Zhao	*Molecular Biology Reports*	2015			✓
S156	Zhao	*Reproductive Biomedicine Online*	2016			✓

No	*First Author*	*Journal*	*Retraction year*	*Method*	*Writing*	*Ethic*
S157	Zheng	*Journal of ECT*	2016	✓		
S158	Zheng	*Journal of ECT*	2016	✓		
S159	Zhgan	*Medicine*	2019			✓
S160	Zhou	*European Journal of Medical Research*	2016			✓
S161	Zhou	*Molecular Biology Reports*	2015			✓
S162	Zhu	*Medicine*	2018			✓
S163	Zhu	*PLoS One*	2018	✓		
S164	Zuo	*Molecular Biology Reports*	2015			✓
S165	Liu	*Aging Clinical and Experimental Research*	2020			✓
S166	Hariyanto	*AIDS Research and Human Retroviruses*	2021			✓
S167	Vlok	*The American Journal of Emergency Medicine*	2020			✓
S168	Wang	*BioMed Research International*	2021	✓		✓
S169	Li	*BioMed Research International*	2020	✓		✓
S170	Sun	*BioMed Research International*	2020	✓		✓
S171	Yang	*BioMed Research International*	2021	✓		✓
S172	Albay	*BMC Neurology*	2020	✓		
S173	Zhong ‡	*Clinical Drug Investigation*	2021	N/A	N/A	N/A
S174	Ferlito	*Clinical Rehabilitation*	2021	✓	✓	
S175	Vorilhon	*European Journal of Clinical Pharmacology*	2021	✓		
S176	Shih	*Experimental and therapeutic medicine*	2021	✓		
S177	Li	*Gastroenterology Research and Practice*	2020	✓		✓
S178	Tian	*Italian Journal of Pediatrics*	2021			✓
S179	Zhou	*Journal of Orthopaedic Surgery and Research*	2021			✓
S180	Hodkinson	*JAMA network open*	2020	✓		
S181	Cai	*Journal of Cellular Biochemistry*	2021	✓		
S182	López-Valverde	*Journal of Clinical Medicine*	2020	✓		
S183	Li	*Journal of Cosmetic Dermatology*	2020			✓
S184	Fan	*Journal of Hypertension*	2021	✓		
S185	Zhang ‡	*Journal of Stroke & Cerebrovascular Diseases*	2021	N/A	N/A	N/A
S186	Guo	*Journal of Zhejiang University-SCIENCE B*	2020	✓		

No	*First Author*	*Journal*	*Retraction year*	*Method*	*Writing*	*Ethic*
S187	Liu	*Medicine*	2020	✓		✓
S188	He	*Medicine*	2020	✓		
S189	Peng	*Medicine*	2020	✓	✓	
S190	Zeng	*Medicine*	2021	✓		
S191	Tan	*Medicine*	2021	✓		
S192	Huang	*Medicine*	2021			✓
S193	Huang	*Medicine*	2021			✓
S194	De Vecchis	*MINERVA CARDIOANGIOLOGICA*	2020			✓
S195	Hussain	*Obesity Research & Clinical Practice*	2021	✓		
S196	Wang	*PLoS One*	2021			✓
S197	Song	*PROCEEDINGS OF THE ROYAL SOCIETY B-BIOLOGICAL SCIENCES*	2021	✓		
S198	Zhang	*Scientific Reports*	2021	✓		

¥ No official announcement or clear reasoning for the retraction.

‡ The reason of retraction: Original research (randomized controlled trials) in their analyses were retracted after the meta-analysis had been published, and authors performed their meta-analysis without cognisance of the concerns with respect to data integrity in the original trials.
