# Supplementary material for: Increasing retractions of meta-analyses publications for methodological flaw
Source: Syst Rev. 2021 Oct 8;10:267. doi: 10.1186/s13643-021-01822-2 (PMC8499503; doi:10.1186/s13643-021-01822-2)

Additional file 5 | Doughnut plot of clarity of reasons for retractions of meta-analyses

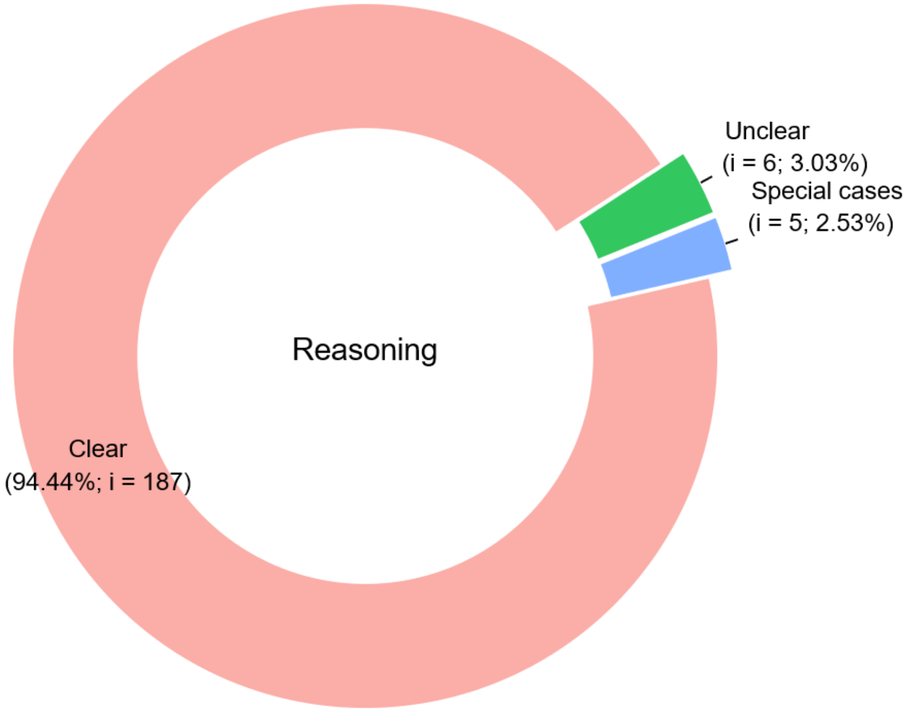

Appendix 6 | Plot of Cochran Q test for three categories of retraction reason

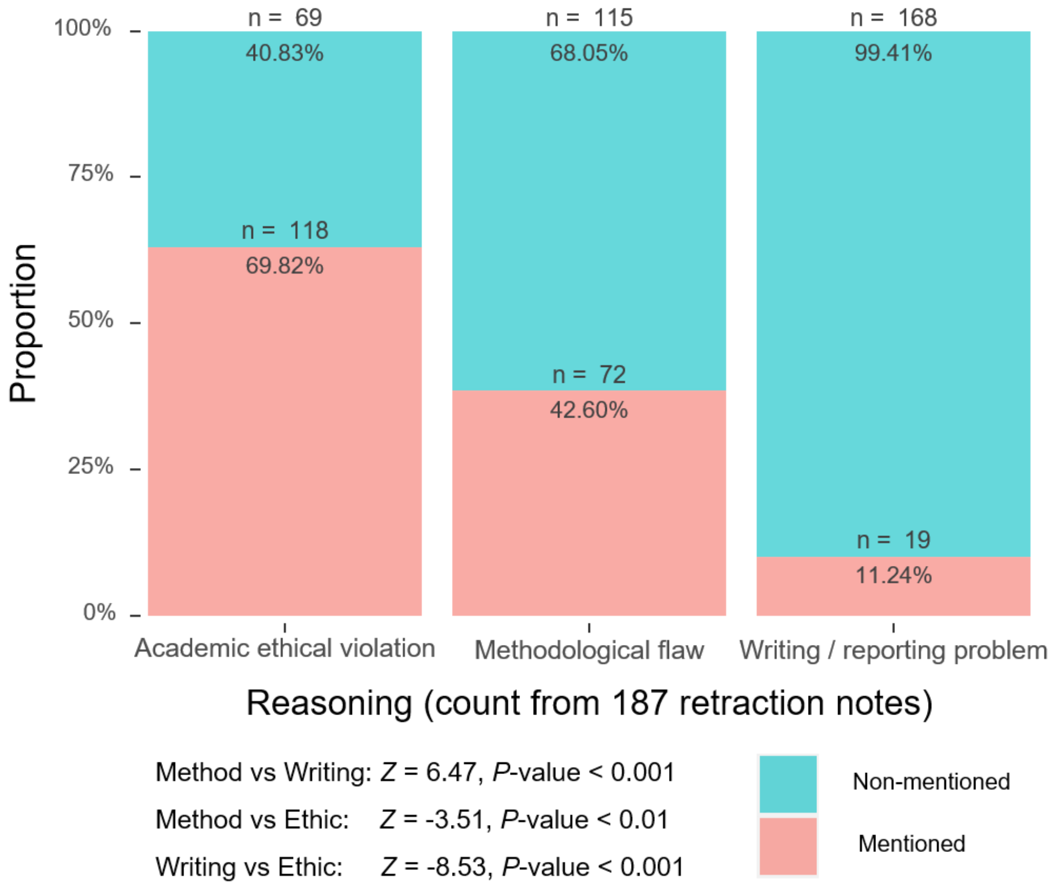

Supplement: Supplementary file 5 — Additional file 5. Doughnut plot of clarity of reasons for retractions of meta-analyses. [file 13643_2021_1822_MOESM5_ESM.pdf]
