# Supplementary material for: Increasing retractions of meta-analyses publications for methodological flaw
Source: Syst Rev. 2021 Oct 8;10:267. doi: 10.1186/s13643-021-01822-2 (PMC8499503; doi:10.1186/s13643-021-01822-2)

Additional file 6 | Plot of Cochran Q test for three categories of retraction reason

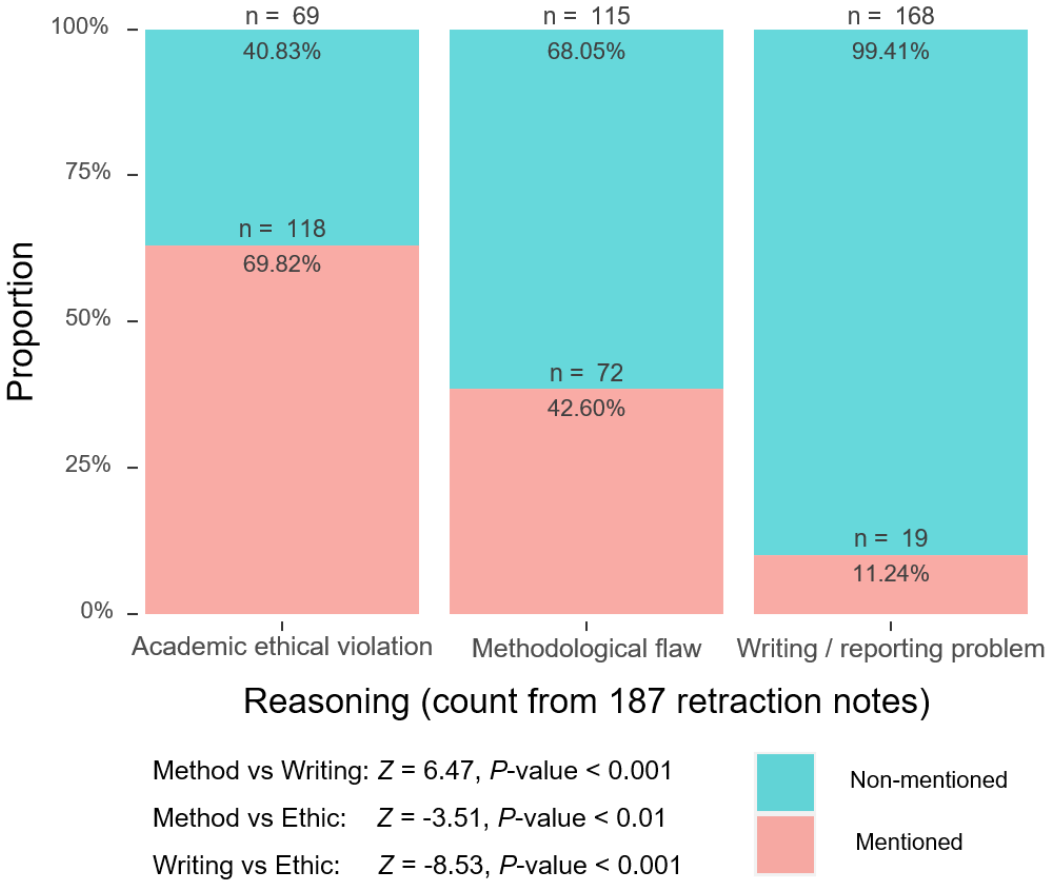

Supplement: Supplementary file 6 — Additional file 6. Plot of Cochran Q test for three categories of retraction reason. [file 13643_2021_1822_MOESM6_ESM.pdf]
